# Pulmonary adverse events following immune checkpoint inhibitors

**DOI:** 10.1097/MCP.0000000000000895

**Published:** 2022-07-16

**Authors:** Paolo Spagnolo, Nazia Chaudhuri, Nicol Bernardinello, Theodoros Karampitsakos, Fotios Sampsonas, Argyrios Tzouvelekis

**Affiliations:** a Respiratory Disease Unit, Department of Cardiac Thoracic, Vascular Sciences and Public Health, University of Padova, Padova, Italy; b Manchester University NHS Foundation. Trust, Manchester, UK; c Department of Respiratory Medicine, University Hospital of Patras, Patras, Greece

**Keywords:** drug-induced lung disease, immune checkpoint inhibitors, interstitial lung disease, nivolumab, pembrolizumab, pneumonia, pulmonary adverse events, pulmonary toxicity

## Abstract

**Purpose of review:**

Immune checkpoint inhibitors (ICIs) have rapidly become a mainstay of cancer treatment. However, immune modulation resulting from checkpoint inhibition can cause inflammation in any organ system, with pneumonitis being one of the most severe immune-related adverse events (irAEs). Here, we review the most recent literature on pulmonary adverse events following ICIs.

**Recent findings:**

Several systematic reviews and meta-analyses of data from trials of antiprogrammed death-1 (PD-1; nivolumab, pembrolizumab), anti-PD-ligand-1 (PD-L1; atezolizumab, avelumab, durvalumab) and anticytotoxic T lymphocyte antigen-4 (CTLA-4; ipilimumab or tremelimumab) in patients with advanced cancer have explored the relative risk and incidence of lung toxicity among different tumor types and therapeutic regimens. They have showed that the incidence of all-grade (1–4) and high-grade (3–4) pneumonitis is significantly higher in nonsmall cell lung cancer (NSCLC) compared with other tumor types. In addition, they have demonstrated that immunotherapy, especially monoimmunotherapy, has a significantly lower risk of irAEs compared to immune-chemotherapy. Treatment for lung cancer, preexisting interstitial lung disease, smoking history and male sex appear to increase the risk for ICI-related pneumonitis.

**Summary:**

Lung toxicity is an uncommon but potentially severe and even fatal complication of ICIs. Timely recognition is critically important but challenging, particularly in patients with lung cancer wherein drug toxicity can mimic disease progression or recurrence.

## INTRODUCTION

Immune checkpoint inhibitors (ICIs) have revolutionized the treatment paradigm of multiple malignancies including nonsmall cell lung cancer (NSCLC). They are immunomodulatory monoclonal antibodies that enhance T cell-mediated cytotoxicity, thereby inducing more effective antitumor immune response [1,2]. The primary targets of checkpoint inhibition include programmed cell death receptor 1 (PD-1; nivolumab, pembrolizumab, cemiplimab and dostarlimab), programmed cell death ligand 1 (PD-L1; atezolizumab, avelumab and durvalumab), and cytotoxic T lymphocyte-associated antigen 4 (CTLA-4; ipilimumab). Their significant clinical benefits, however, can be hampered by the development of discrete toxicities referred to as immune-related adverse events (irAEs), which are caused by activation of the immune system and can cause inflammation within any organ. IrAEs include, but are not limited to, gastrointestinal, cutaneous, hepatic, and endocrine complications, although the most frequent severe irAE, is pneumonitis, which occurs with an overall incidence of 2.7% with monotherapy and 6.6% with combination therapy [3–6]. The pathogenesis of ICI-induced pneumonitis remains unclear but immune hyperactivation may be a contributing factor by causing alveolitis and endothelitis. In particular, increased CD4+ lymphocytes and decreased Tregs activity have been described in the bronchoalveolar lavage fluid (BALF) of patients with ICI-induced pneumonitis indicating dysregulated T cell activity against cross-antigens expressed in normal alveolar epithelial as well as in cancer cells [7]. As with other drug-induced adverse events, ICI-related pneumonitis is a diagnosis of exclusion, which implies that alternative diagnoses, mainly infection and recurrence/progression of cancer, need to be excluded [8^▪^]. Most irAEs are mild to moderate in severity, although severe and even fatal events have also been reported [1,9], and occur idiosyncratically, generally within weeks to 3 months after treatment initiation, but they can also appear as late as 1 year after treatment termination [10^▪^]. Overall, ICIs present a favorable benefit-to-risk profile compared to conventional chemotherapy, with PD-L1 inhibitors being less toxic than PD-1 inhibitors (probably because they do not block the PD-L2 and PD-1 interaction), and high-grade toxicity being more common with CTLA-4/PD-1 or PD-L1 combination than with anti-PD-1 monotherapy.

The last few years have witnessed an exponential increase in the number of clinical trials investigating the role of ICIs in multiple malignancies. In this article, we summarize the most recent literature on pulmonary complications of ICIs, a phenomenon that is likely to become more common in the future, owing to the longer median survival of cancer patients. 

**Box 1:**
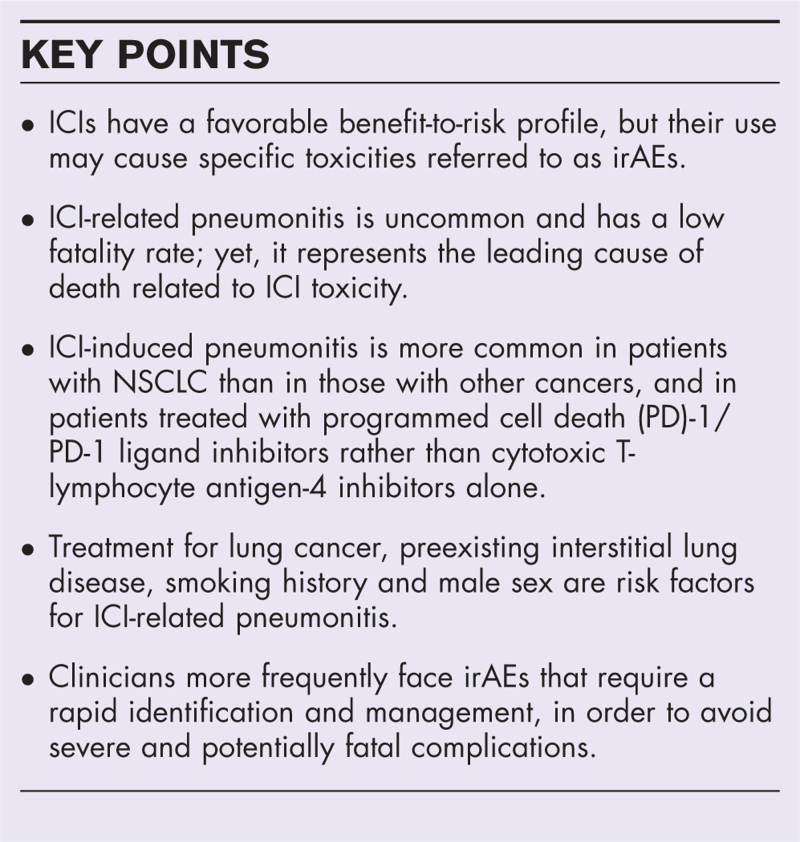
no caption available

## NIVOLUMAB

Nivolumab has shown consistent efficacy in prolonging survival in several advanced malignancies, including melanoma [11], NSCLC [12,13], Hodgkin's lymphoma [14], renal cell carcinoma [15] and urethral carcinoma [16] with ever expanding indications [17], either as a single agent or in combination with other ICIs and anticancer therapies. The incidence of pulmonary adverse events attributed to nivolumab can be a challenge to accurately quantify due to the heterogeneity of published studies (Table 1). Indeed, studies vary in geographical patient populations, underlying malignancies being treated, comorbidities and the presence of concomitant therapies, all of which can impact on the susceptibility of developing pulmonary toxicity. Data from clinical trials, postmarketing authorization studies and adverse event monitoring databases can provide us with the most accurate information on these adverse events.

**Table 1:** Pattern of immune checkpoint inhibitor-induced pulmonary adverse events

*Nivolumab*	Interstitial lung disease, pneumonitis, OP, AFOP, sarcoidosis/sarcoid-like reaction, pleural effusion, reactivation of pulmonary infection (tuberculosis, aspergillosis), lung cavitation, asthma.
*Pembrolizumab*	Pneumonitis (increased risk of high-grade pneumonitis compared with nivolumab), pleural effusion, sarcoidosis/sarcoid-like reactions, reactivation of tuberculosis, AFOP, OP, eosinophilic pneumonia, ARDS, lung cavitation, asthma exacerbation, alveolar hemorrhage.
*Nivolumab/ipilimumab*	Pneumonitis (increased risk of all-grade pneumonitis compared with PD-1 inhibitor monotherapy), sarcoidosis.
*Atezolimumab*	Pneumonitis (overall, PD-L1 inhibitors have a lower incidence of all-grade pneumonitis compared with PD-1 inhibitors).
*Durvalumab*	Pneumonitis
*Avelumb*	Pneumonitis, sarcoid-like reaction

AFOP, Acute Fibrinous Organizing Pneumonia; ARDS, Acute Respiratory Distress Syndrome; OP, organizing pneumonia; PD-1, Programmed Death-1; PD-L1, Programmed Death-Ligand 1.

A meta-analysis containing ten trials of nivolumab therapy in patients with NSCLC showed a heterogeneous range of incidence of any grade and greater than grade 3 pneumonitis, ranging from 2.79–7.75% to 0.37–3.42%, respectively, with the highest rates being observed among treatment naïve patients [18]. Furthermore, unlike adverse events, such as fatigue, nausea, diarrhea, and neutropenia where nivolumab fared better than conventional chemotherapy [18], the odds ratio of immune-related all-grade and high-grade pneumonitis was 6.29 [95% confidence interval (CI): 2.67–16.75] and 5.95 (95% CI: 2.35–17.29), respectively for nivolumab compared to conventional chemotherapy, with combination with ipilimumab (OR 14.82) being associated with a higher risk of pneumonitis [19]. In a further meta-analysis of 46 studies of 12 808 patients, the incidence of adverse events varied according to the drug type and cancer being treated. Any grade irAEs with all doses of nivolumab was 4.8% (95% CI: 4.01–5.56%) for all cancer patients. Cutaneous, endocrine, and gastrointestinal irAEs were the commonest at 25.47% (95% CI: 20.5–31.2%), 10.2% (95% CI: 8.32–12.53%) and 13.6% (95% CI: 10.91–16.84%), respectively, for all cancers. Pulmonary adverse events were lower at 4.75% (95% CI: 3.52–6.38%). Nivolumab-induced pulmonary adverse events of all grades occurred more frequently during NSCLC (3.88%, 95% CI: 2.57–5.82%) and renal cell cancer treatment (7.19%, 95% CI: 4.54–11.21%) than during melanoma treatment (1.77%, 95% CI: 1.09–2.84%) [20]. Notably, interstitial lung disease (ILD) onset occurred earlier in NSCLC compared to melanoma (median 2.1 versus 5.2 months respectively *P* = 0.02) [21]. This is a consistent finding in other studies [22]. In a meta-analysis of 35 trials in advanced melanoma, the incidence of all grade pneumonitis was much lower than other cancers at 1.4% [23^▪^], thus highlighting that the incidence of pulmonary adverse events is influenced by the underlying cancer being treated. The mechanisms accounting for this difference can only be speculated upon; one hypothesis is that NSCLC patients have a pattern of risk factors that is different from individuals with melanoma, and includes smoking history, male sex, older age, and ILD [21].

The incidence of adverse events is also influenced by the population being studied. Contrary to the above study where cutaneous, endocrine, and gastrointestinal adverse events were commonest [20], in 21 studies of 6173 patients with varied cancers, the commonest serious adverse events were pneumonitis and ILD at 8.2 and 3.6% respectively and differed according to cancer type with lung and gastric cancer predominating [24]. Similarly, in post marketing Japanese surveillance studies in NSCLC [25], and head and neck cancer patients [26], the commonest adverse events were ILD at 6.4 and 4.1% respectively [25,26]. Nivolumab-induced ILD is the commonest adverse events reported in Japanese and Chinese populations compared to studies in other geographic groups [27].

Understanding which group of patients are at highest risk of developing pulmonary adverse events can aid clinicians and patients to have informed discussions about the risks versus benefits of immunotherapy (Table 2). In one study, risk factors for nivolumab-induced ILD included preexisting ILD (25.3 versus 8.5%), abnormal radiological findings prior to nivolumab therapy (16 versus 7.8%) and smoking history (10.7 versus 6.2%) [25]. Furthermore, in a retrospective single center Japanese study of 188 patients diagnosed with head and neck cancer, NSCLC and gastric cancer, pneumonitis rates were also higher in those with preexisting ILD compared to those without ILD (35 versus 5.4%). Multivariate analysis revealed pre-existing ILD and male gender as independent risk factors for developing pneumonitis with OR of 5.92 (95% CI: 2.07–18.54; *P* = 0.0008) and 5.58; (95% CI: 1.01–104.40; *P* = 0.049), respectively [28^▪^]. Similar risk factors were found in other studies in NSCLC, leading to the hypothesis that smoking history and pre-existing lung disease may be pathogenetic drivers of pulmonary toxicity [29].

**Table 2:** Potential risk factors for immune checkpoint inhibitor-induced pulmonary adverse events

Combination of ICIs (especially PD-L1 and CTLA-4)Combination of EGFR-TKIs and ICIsConcomitant or sequential ICI and thoracic radiationPrior thoracic radiationPrior chemotherapyPreexisting obstructive lung diseases (asthma, COPD)Preexisting interstitial lung diseasePreexisting connective tissue diseaseImmunosuppressive treatmentPoor performance status (ECOG PS) of ≥ 2NSCLC compared with melanomaCertain histologies (adenocarcinoma compared to other NSCLC histologic subtypes)Smoking history

COPD, Chronic Obstructive Pulmonary Disease; CTLA-4, Cytotoxic T-Lymphocyte Antigen-4; ECOG PS, Eastern Cooperative Oncology Group performance status; EGFR, Epidermal Growth Factor Receptor; ICIs, Immune Checkpoint Inhibitors; NSCLC, Non-Small Cell Lung Cancer; PD-L1, Programmed Death-Ligand 1; TKIs, Tyrosine Kinase Inhibitors.

**FIGURE 1:**
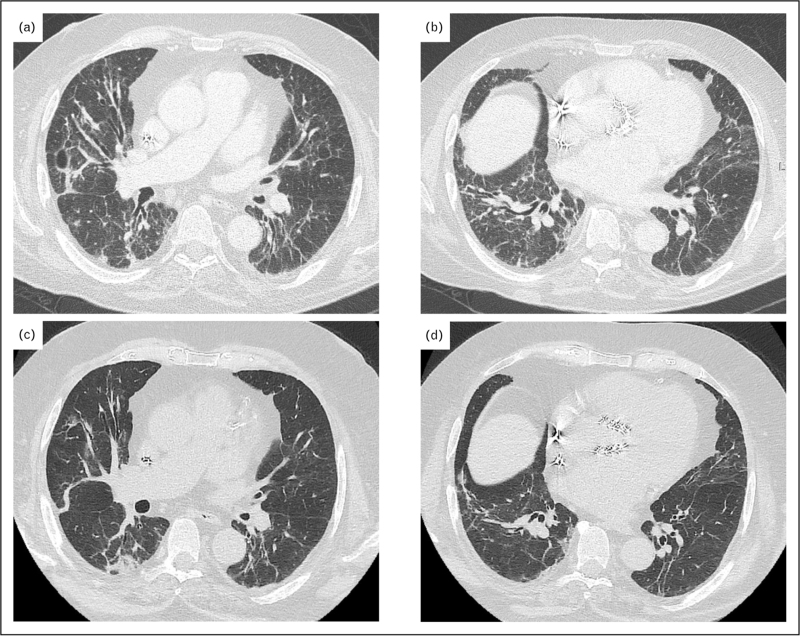
Organizing pneumonia/nonspecific interstitial pneumonia pattern in a 79-year-old man undergoing nivolumab therapy for advanced melanoma. (a, b) Axial chest computed tomography images obtained 3 months after nivolumab therapy show multifocal mid- and lower-lung linear opacities and airspace consolidations, a finding consistent with an organizing pneumonia pattern of pneumonitis. (c, d) Axial chest computed tomography images obtained 2 years later after withholding nivolumab and administering systemic steroid therapy show significant residual disease.

Radiologically, ground glass and consolidation are the commonest features of nivolumab pulmonary adverse events [21], with organizing pneumonia [21,30], nonspecific interstitial pneumonia [30] and hypersensitivity pneumonitis [21] being the commonest patterns observed (Fig. 1). Bronchoalveolar lavage when performed, can be helpful to exclude alternative diagnoses, mainly infection, and one study has reported a lymphocytosis above 15% in most patients [21]. Although not evidence based, steroid therapy is the mainstay of therapy coupled with treatment interruption [30,31]. The National Comprehensive Cancer Network provides guidance on the management of immunotherapy pneumonitis [32]. Most patients recover or improve after treatment cessation and/or steroid therapy with rates varying from 70 to 87% [21,30,31], highlighting the importance of early identification, diagnosis, and management of nivolumab-related pulmonary adverse events. Retreatment with nivolumab after the development of pneumonitis is a challenging issue and warrants further investigation regarding safety and outcomes [33].

## PEMBROLIZUMAB

Over the last few years, pembrolizumab has been used in multiple solid and nonsolid malignancies as first, subsequent line or maintenance treatment, alone or in combination with chemo/radiotherapy or other ICIs [34]. Pembrolizumab-induced pneumonitis of various grades has been reported with both pembrolizumab monotherapy or in combination with standard chemo/radiotherapy or other ICIs in patients with NSCLC, with incidence ranging from 2.8% to 28% [35,36^▪^,^37^]. Pembrolizumab is approved for advanced NSCLC either as first-line or second line monotherapy based on the phase III KEYNOTE-024 [38] and KEYNOTE-010 trials [39]. Any grade and severe (grades 3–5) pneumonitis were reported in 5.9% and 2.6% of patients in KEYNOTE-010 and 7.8% and 2.6% of patients in KEYNOTE-024, respectively. In addition, pembrolizumab is approved in combination with (nab)paclitaxel and platinum for metastasized squamous NSCLC based on the results of the phase III KEYNOTE-407 trial [40]. Notably, this combination was associated with one of the highest prevalence of reported irAEs, with pneumonitis being particularly prevalent (any grade pneumonitis 6.5%, and greater than grade 3 pneumonitis 2.5%, including one patient with grade 5 pneumonitis). Overall, pneumonitis of any grade has been reported in 3.4% of patients treated with pembrolizumab for various cancers and in 8.2% of patients with advanced NSCLC treated with pembrolizumab as single agent first-line treatment, with severe pneumonitis occurring in 1.3% and 3.2% of cases, respectively. These numbers may be higher in patients with preexisting ILD, but this is unclear as these patients are generally excluded from clinical trials of ICIs.

Pneumonitis usually develops within 3 months from pembrolizumab administration; yet, more acute events have been described, particularly in patients with melanoma, with a median time of onset of only 10 days [41]. Increased body mass index, concurrent chemotherapy, epidermal growth factor receptor (EGFR) mutations, increased age, preexisting ILD, tumor infiltration, and concurrent obstruction of central airways are risk factors for early (less than 3 months) occurrence of pneumonitis [42,43]. Pulmonary adverse events in lung cancer patients may also involve the pleura, leading in some cases to pleural effusions that may be misdiagnosed as disease relapse [44^▪▪^]. Radiological patterns of lung disease include hypersensitivity pneumonitis, acute interstitial pneumonitis, organizing pneumonia, nonspecific interstitial pneumonia, traction bronchiectasis, radiation recall pneumonitis (e.g., delayed inflammatory reaction in a previously irradiated lung parenchyma), bronchiolitis and, rarely, acute fibrinous organizing pneumonia and lipoid pneumonia [45,46]. Pembrolizumab monotherapy has been associated more frequently with acute pulmonary events, including ICI-related pneumonitis and pulmonary embolism requiring hospitalization, compared to other ICIs [45]. Of note, only 12.9% of these events were solely pneumonitis [45]. In addition, concomitant administration of pembrolizumab with other ICIs is strongly associated with thromboembolic events in melanoma and metastatic gastric cancer patients [47]. On the contrary, pneumonia due to severe immunodepression is the most common infectious complications of pembrolizumab [48].

A recent meta-analysis of 14 randomized controlled trials (RCTs) showed that pembrolizumab is the second cause of severe pneumonitis, the first being nivolumab [49^▪▪^]; yet, another meta-analysis comprising 9318 patients in 18 phase III RCTs reported that pembrolizumab exerted the greatest risk for pneumonitis development (pooled relative risk = 3.12; 95% CI, 2.06–4.73; *P* < 0.00001) [50]. Pembrolizumab-induced pneumonitis is also frequently encountered in nonlung cancer patients, including patients with mesothelioma (8%) [51], melanoma and myelodysplastic disorders, although the vast majority of cases were of mild severity [52]. Conversely, pembrolizumab-induced pneumonitis is uncommon in renal cancer and multiple myeloma patients [53,54]. Of note, pembrolizumab-induced lung injury in nonlung cancer patients is characterized by different radiological patterns compared to lung cancer, including ground glass opacities mainly involving the lower lobes, whereas organizing pneumonia, reverse halo patterns, bronchiectasis and fibrotic changes have been less frequently described [46]. Interestingly, pembrolizumab irAEs have been associated with increased progression-free survival (PFS) in NSCLC patients, implying an exaggerated inflammatory response with potential anticancer effects [55]; yet, this remains to be further elucidated.

Pre-existing ILD represents an independent risk factor for severe immune-related pneumonitis [56]. Nevertheless, these findings by no means reduce the favorable outcomes of pembrolizumab in patients with lung cancer irrespective of concomitant ILD [56,57]. During the severe acute respiratory syndrome coronavirus 2 (SARS COV-2) pandemic, combination treatment of pembrolizumab with other ICIs, was an independent risk factor for hospital admission, along with Eastern Cooperative Oncology Group scores ≥2 and the presence of COVID-19 symptoms, but was not related to death [58]. The risk of exposing NSCLC patients to SARS COV-2 has led to extensive interval doses with many ICIs, including pembrolizumab, with comparable PFS to standard dose but with minor frequency of pneumonitis [59].

ICI-induced pneumonitis is managed according to a grading system ranging from 1–4, with grades 3–4 requiring steroid treatment and hospitalization [60]. Microbiological, cellular and molecular analysis of BALF of patients with confirmed pembrolizumab-induced pneumonitis revealed a lymphocytic and interleukin-6 predominance, providing useful pathogenetic and therapeutic insights [61^▪^]. Currently there are no biomarkers specific for ICIs-induced pneumonitis, although Krebs von den Lungen-6, which is produced by type II pneumocytes and airway epithelial cells in response to damage, has been effectively used to differentiate between immune-related pneumonitis and infection [46]. Yet, further studies are needed to support its use in clinical practice.

## ATEZOLIZUMAB

Atezolizumab-induced grade 1–2 pneumonitis occurs in about 1% of patients treated for advanced NSCLC, but is much rarer in patients with advanced urothelial carcinoma and various other malignancies [62]. Grade 3 pneumonitis has been reported in 0.6% and 2% of patients with advanced NSCLC in the BIRCH (Study of Atezolizumab in Participants With PD-L1 Positive Locally Advanced or Metastatic Non–Small-Cell Lung Cancer) [63] and POPLAR (Study of Atezolizumab versus docetaxel for patients with previously treated NSCLC) studies [64], respectively. Similarly, in the OAK (Atezolizumab Versus Docetaxel in Patients with Previously Treated Non–Small-Cell Lung Cancer) study, the overall incidence of pneumonitis was 1%, with no grade 4 events being reported [65].

## AVELUMAB

Pneumonitis is an uncommon complication of avelumab treatment. In a phase II, open-label study that assessed avelumab treatment in patients with stage IV Merkel cell carcinoma that had progressed after cytotoxic chemotherapy, only one patient developed pneumonia [66]. Sarcoid-like reaction following avelumab treatment has also been reported [67].

## DURVALUMAB

The PACIFIC was a phase III study that compared durvalumab as consolidation therapy with placebo in patients with stage III NSCLC who did not have disease progression after two or more cycles of platinum-based chemoradiotherapy [68]. Pneumonitis or radiation pneumonitis of any grade occurred in 33.9% of patients in the durvalumab arm and 24.8% of patients in the placebo arm, whereas pneumonitis or radiation pneumonitis of grade 3 or 4 occurred in 3.4% of patients who received durvalumab and 2.6% of patients who received placebo. Pneumonia of any grade occurred in 13.1% and 7.7%, and pneumonia of grade 3 or 4 occurred in 4.4% and 3.8% of patients in the durvalumab and placebo group, respectively. The ATLANTIC study assessed the effect of durvalumab treatment in three cohorts of patients with NSCLC defined by EGFR/anaplastic lymphoma kinase status and tumor expression of PD-L1 [69]. Grade 3 to 4 pneumonitis occurred in 4/444 patients (1%).

## NIVOLUMAB/IPILIMUMAB

The incidence of pneumonitis during nivolumab/ipilimumab combination therapy was assessed in three studies of patients with melanoma [70–72]. Compared with PD-1 inhibitor monotherapy, nivolumab/ipilimumab combination therapy was associated with a significantly higher incidence of all-grade and grade 3 or higher pneumonitis. In addition, the only pneumonitis-related death occurred in the nivolumab/ipilimumab combination arm. Similarly, in a study that assessed the safety and efficacy of nivolumab and nivolumab/ipilimumab in patients with SCLC, the only treatment-related death was reported in the nivolumab/ipilimumab group [73]. Cui and colleagues conducted a meta-analysis of 12 trials that evaluated nivolumab/ipilimumab combination therapy and confirmed that, compared with nivolumab monotherapy, nivolumab/ipilimumab combination is associated with a significantly higher risk of all-grade (OR:3.54) and high-grade pneumonitis (OR:2.35) [74], suggesting an additive toxic effect of the two drugs on the lung. Nivolumab/ipilimumab combination therapy has also been associated with the development of pulmonary and cutaneous sarcoidosis in a patient with recurrent melanoma [75]. Such cases, which are likely to be underestimated, may represent a diagnostic dilemma, as sarcoidosis lesions can mimic disease progression or metastatic disease.

### Retreatment with immune checkpoint inhibitor following a previous episode of pneumonitis

The possibility to reintroduce immunotherapy following ICI-induced pneumonitis depends mostly on the severity of the previous pulmonary adverse event. Although there are no clear guidelines in this regard, re-challenge could be considered in Grade 1–2 pneumonitis in the presence of resolution or significant clinical/radiographic improvement, whereas for Grade 3 pneumonitis, rechallenge should be assessed on a case-by-case basis and only once symptoms and radiographic abnormalities have resolved, along with close monitoring. For Grade 4 pneumonitis, permanent ICI discontinuation is recommended.

## CONCLUSION

Lung toxicity secondary to ICI treatment is an uncommon but potentially severe complication that usually occurs within the first few months of treatment. The use of ICIs has rapidly expanded well beyond melanoma and with wider use and the development of a new generation of such drugs additional adverse events are likely to ensue. Early recognition and management of ICI-related adverse events are therefore critically important. Additional studies are needed to clarify the mechanisms through which immunotherapy causes a spectrum of immune-related events.

## Acknowledgements

*None.*

### Financial support and sponsorship

*None.*

### Conflicts of interest

*There are no conflicts of interest.*

## REFERENCES AND RECOMMENDED READING

Papers of particular interest, published within the annual period of review, have been highlighted as:

▪ of special interest
▪▪ of outstanding interest

## References

[1] Topalian SL Hodi FS Brahmer JR. Safety, activity, and immune correlates of anti-PD-1 antibody in cancer. N Engl J Med 2012; 366:2443–2454. 22658127 10.1056/NEJMoa1200690 PMC3544539

[2] Brahmer JR Tykodi SS Chow LQ. Safety and activity of anti-PD-L1 antibody in patients with advanced cancer. N Engl J Med 2012; 366:2455–2465. 22658128 10.1056/NEJMoa1200694 PMC3563263

[3] Nishino M Giobbie-Hurder A Hatabu H. Incidence of programmed cell death 1 inhibitor-related pneumonitis in patients with advanced cancer: a systematic review and meta-analysis. JAMA Oncol 2016; 2:1607–1616. 27540850 10.1001/jamaoncol.2016.2453

[4] Khunger M Rakshit S Pasupuleti V. Incidence of pneumonitis with use of programmed death 1 and programmed death-ligand 1 inhibitors in non-small cell lung cancer: a systematic review and meta-analysis of trials. Chest 2017; 152:271–281. 28499515 10.1016/j.chest.2017.04.177

[5] Chen X Wu X Wu H. Camrelizumab plus gemcitabine and oxaliplatin (GEMOX) in patients with advanced biliary tract cancer: a single-arm, open-label, phase II trial. J Immunother Cancer 2020; 8:e001240. 33172881 10.1136/jitc-2020-001240 PMC7656907

[6] Liu T Jin B Chen J. Comparative risk of serious and fatal treatment-related adverse events caused by 19 immune checkpoint inhibitors used in cancer treatment: a network meta-analysis. Ther Adv Med Oncol 2020; 12:1758835920940927. 32774474 10.1177/1758835920940927 PMC7394035

[7] Suresh K Naidoo J Zhong Q. The alveolar immune cell landscape is dysregulated in checkpoint inhibitor pneumonitis. J Clin Invest 2019; 129:4305–4315. 31310589 10.1172/JCI128654 PMC6763233

[8▪] Spagnolo P Bonniaud P Rossi G. Drug-induced interstitial lung disease. Eur Respir J 2022; 2102776 doi:10.1183/13993003.02776-2021. 35332071 10.1183/13993003.02776-2021

[9] Garon EB Rizvi NA Hui R. Pembrolizumab for the treatment of nonsmall-cell lung cancer. N Engl J Med 2015; 372:2018–2028. 25891174 10.1056/NEJMoa1501824

[10▪] Skribek M Rounis K Tsakonas G Ekman S. Complications following novel therapies for nonsmall cell lung cancer. J Intern Med 2022; 291:732–754. 35032058 10.1111/joim.13445

[11] Weber JS D’Angelo SP Minor D. Nivolumab versus chemotherapy in patients with advanced melanoma who progressed after anti-CTLA-4 treatment (CheckMate 037): a randomised, controlled, open-label, phase 3 trial. Lancet Oncol 2015; 16:375–384. 25795410 10.1016/S1470-2045(15)70076-8

[12] Gettinger SN Redman MW Bazhenova L. Nivolumab plus ipilimumab vs nivolumab for previously treated patients with stage IV squamous cell lung cancer: the lung-MAP S1400I Phase 3 randomized clinical trial. JAMA Oncol 2021; 7:1368–1377. 34264316 10.1001/jamaoncol.2021.2209 PMC8283667

[13] Borghaei H Gettinger S Vokes EE. Five-year outcomes from the randomized, Phase III Trials CheckMate 017 and 057: nivolumab versus docetaxel in previously treated non-small-cell lung cancer. J Clin Oncol 2021; 39:723–733. 33449799 10.1200/JCO.20.01605 PMC8078445

[14] Ansell SM Lesokhin AM Borrello I. PD-1 blockade with nivolumab in relapsed or refractory Hodgkin's lymphoma. N Engl J Med 2015; 372:311–319. 25482239 10.1056/NEJMoa1411087 PMC4348009

[15] Choueiri TK Powles T Burotto M. Nivolumab plus cabozantinib versus sunitinib for advanced renal-cell carcinoma. N Engl J Med 2021; 384:829–841. 33657295 10.1056/NEJMoa2026982 PMC8436591

[16] Bajorin DF Witjes JA Gschwend JE. Adjuvant nivolumab versus placebo in muscle-invasive urothelial carcinoma. N Engl J Med 2021; 384:2102–2114. 34077643 10.1056/NEJMoa2034442 PMC8215888

[17] Janjigian YY Shitara K Moehler M. First-line nivolumab plus chemotherapy versus chemotherapy alone for advanced gastric, gastro-oesophageal junction, and oesophageal adenocarcinoma (CheckMate 649): a randomised, open-label, phase 3 trial. Lancet 2021; 398:27–40. 34102137 10.1016/S0140-6736(21)00797-2 PMC8436782

[18] Peng TR Tsai FP Wu TW. Indirect comparison between pembrolizumab and nivolumab for the treatment of nonsmall cell lung cancer: a meta-analysis of randomized clinical trials. Int Immunopharmacol 2017; 49:85–94. 28554108 10.1016/j.intimp.2017.05.019

[19] Huang Y Fan H Li N Du J. Risk of immune-related pneumonitis for PD1/PD-L1 inhibitors: systematic review and network meta-analysis. Cancer Med 2019; 8:2664–2674. 30950194 10.1002/cam4.2104 PMC6536966

[20] Wang PF Chen Y Song SY. Immune-related adverse events associated with anti-PD-1/PD-L1 treatment for malignancies: a meta-analysis. Front Pharmacol 2017; 8:730. 29093678 10.3389/fphar.2017.00730 PMC5651530

[21] Delaunay M Cadranel J Lusque A. Immune-checkpoint inhibitors associated with interstitial lung disease in cancer patients. Eur Respir J 2017; 50:1700050. 28798088 10.1183/13993003.00050-2017

[22] Ma K Lu Y Jiang S. The relative risk and incidence of immune checkpoint inhibitors related pneumonitis in patients with advanced cancer: a meta-analysis. Front Pharmacol 2018; 9:1430. 30618738 10.3389/fphar.2018.01430 PMC6297260

[23▪] Almutairi AR McBride A Slack M. Potential immune-related adverse events associated with monotherapy and combination therapy of ipilimumab, nivolumab, and pembrolizumab for advanced melanoma: a systematic review and meta-analysis. Front Oncol 2020; 10:91. 32117745 10.3389/fonc.2020.00091 PMC7033582

[24] Zhao B Zhao H Zhao J. Serious adverse events and fatal adverse events associated with nivolumab treatment in cancer patients: Nivolumab-related serious/fatal adverse events. J Immunother Cancer 2018; 6:101. 30285872 10.1186/s40425-018-0421-z PMC6171173

[25] Yamamoto N Nakanishi Y Gemma A. Real-world safety of nivolumab in patients with nonsmall-cell lung cancer in Japan: Postmarketing surveillance. Cancer Sci 112:4692–4701. 34431585 10.1111/cas.15117 PMC8586674

[26] Tahara M Kiyota N Nibu KI. Realworld safety and effectiveness of nivolumab for recurrent or metastatic head and neck cancer in Japan: a postmarketing surveillance. Int J Clin Oncol 2021; 26:1619–1627. 34110532 10.1007/s10147-021-01949-1 PMC8364900

[27] Shi Y Fang J Zhou C. Immune checkpoint inhibitor-related adverse events in lung cancer: Real-world incidence and management practices of 1905 patients in China. Thorac Cancer 2022; 13:412–422. 34935288 10.1111/1759-7714.14274 PMC8807338

[28▪] Yamaguchi T Shimizu J Hasegawa T. Preexisting interstitial lung disease is associated with onset of nivolumab-induced pneumonitis in patients with solid tumors: a retrospective analysis. BMC Cancer 2021; 21:924. 34399710 10.1186/s12885-021-08661-3 PMC8369733

[29] Kanai O Kim YH Demura Y. Efficacy and safety of nivolumab in nonsmall cell lung cancer with preexisting interstitial lung disease. Thorac Cancer 2018; 9:847–855. 29782069 10.1111/1759-7714.12759 PMC6026605

[30] Kato T Masuda N Nakanishi Y. Nivolumab-induced interstitial lung disease analysis of two phase II studies patients with recurrent or advanced nonsmall-cell lung cancer. Lung Cancer 2017; 104:111–118. 28212992 10.1016/j.lungcan.2016.12.016

[31] Sata M Sasaki S Oikado K. Treatment and relapse of interstitial lung disease in nivolumab-treated patients with nonsmall cell lung cancer. Cancer Sci 2021; 112:1506–1513. 33125784 10.1111/cas.14715 PMC8019226

[32] Thompson JA Schneider BJ Brahmer J. Management of immunotherapy-related toxicities, version 1.2019. J Natl Compr Cancer Network 2019; 17:255–289. 10.6004/jnccn.2019.0013 30865922

[33] Nishino M Ramaiya NH Awad MM. PD-1 inhibitor-related pneumonitis in advanced cancer patients: radiographic patterns and clinical course. Clin Cancer Res 2016; 22:6051–6060. 27535979 10.1158/1078-0432.CCR-16-1320 PMC5161686

[34] Khoja L Butler MO Kang SP. Pembrolizumab. J Immunother Cancer 2015; 3: 10.1186/s40425-015-0078-9 PMC4539882 26288737

[35] Fujimoto D Miura S Yoshimura K. A real-world study on the effectiveness and safety of pembrolizumab plus chemotherapy for nonsquamous NSCLC. JTO Clin Res Rep 2021; 3:100265. 35146460 10.1016/j.jtocrr.2021.100265 PMC8819387

[36▪] Zheng X Tao G Sun S. Adverse events of different PD-1 inhibitors in lung cancer patients: a real-world study. Ann Transl Med 2022; 10:183. 35280395 10.21037/atm-21-6899 PMC8908189

[37] Hasegawa S Ikesue H Nakao S. Analysis of immune-related adverse events caused by immune checkpoint inhibitors using the Japanese Adverse Drug Event Report database. Pharmacoepidemiol Drug Saf 2020; 29:1279–1294. 32869941 10.1002/pds.5108 PMC7692939

[38] Reck M Rodríguez-Abreu D Robinson AG. Pembrolizumab versus chemotherapy for PD-L1-positive non-small-cell lung cancer. N Engl J Med 2016; 375:1823–1833. 27718847 10.1056/NEJMoa1606774

[39] Herbst RS Baas P Kim DW. Pembrolizumab versus docetaxel for previously treated, PD-L1-positive, advanced nonsmall-cell lung cancer (KEYNOTE-010): a randomised controlled trial. Lancet 2016; 387:1540–1550. 26712084 10.1016/S0140-6736(15)01281-7

[40] Paz-Ares L Luft A Vicente D. Pembrolizumab plus chemotherapy for squamous non-small-cell lung cancer. N Engl J Med 2018; 379:2040–2051. 30280635 10.1056/NEJMoa1810865

[41] Suo A Chan Y Beaulieu C. Anti-PD1-induced immune-related adverse events and survival outcomes in advanced melanoma. Oncologist 2020; 25:438–446. 32048768 10.1634/theoncologist.2019-0674 PMC7216458

[42] Huang Y Soon YY Aminkeng F. Risk factors for immune-related adverse events from anti-PD-1 or anti-PD-L1 treatment in an Asian cohort of nonsmall cell lung cancer patients. Int J Cancer 2022; 150:636–644. 34562273 10.1002/ijc.33822

[43] Yamaguchi T Shimizu J Oya Y. Risk factors for pneumonitis in patients with nonsmall cell lung cancer treated with immune checkpoint inhibitors plus chemotherapy: a retrospective analysis. Thorac Cancer 2022; 13:724–731. 35044093 10.1111/1759-7714.14308 PMC8888158

[44▪▪] Berti A Bortolotti R Dipasquale M. Meta-analysis of immune-related adverse events in phase 3 clinical trials assessing immune checkpoint inhibitors for lung cancer. Crit Rev Oncol Hematol 2021; 162:103351. 33989769 10.1016/j.critrevonc.2021.103351

[45] Smith DA Radzinsky E Tirumani SH. Findings on chest CT performed in the emergency department in patients receiving immune checkpoint inhibitor therapy: single-institution 8-year experience in 136 patients. AJR Am J Roentgenol 2020; 217:613–622. 33295801 10.2214/AJR.20.24758

[46] Nobashi TW Nishimoto Y Kawata Y. Clinical and radiological features of immune checkpoint inhibitor-related pneumonitis in lung cancer and nonlung cancers. Br J Radiol 2020; 93:20200409. 32783627 10.1259/bjr.20200409 PMC8519648

[47] Sussman TA Li H Hobbs B. Incidence of thromboembolism in patients with melanoma on immune checkpoint inhibitor therapy and its adverse association with survival. J Immunother Cancer 2021; 9:e001719. 33436486 10.1136/jitc-2020-001719 PMC7805375

[48] Bavaro DF Pizzutilo P Catino A. Incidence of infections and predictors of mortality during checkpoint inhibitor immunotherapy in patients with advanced lung cancer: a retrospective cohort study. Open Forum Infect Dis 2021; 8:ofab187. 34141817 10.1093/ofid/ofab187 PMC8204890

[49▪▪] Gu J Shi L Jiang X. Severe immune-related adverse events of immune checkpoint inhibitors for advanced nonsmall cell lung cancer: a network meta-analysis of randomized clinical trials. Cancer Immunol Immunother 2022; doi: 10.1007/s00262-022-03140-5. [Online ahead of print]. 10.1007/s00262-022-03140-5 PMC10992828 35124713

[50] Wang Y Kong D Wang C. A systematic review and meta-analysis of immune-related adverse events of anti-PD-1 drugs in randomized controlled trials. Technol Cancer Res Treat 2020; 19:1533033820967454. 33084525 10.1177/1533033820967454 PMC7588773

[51] Yap TA Nakagawa K Fujimoto N. Efficacy and safety of pembrolizumab in patients with advanced mesothelioma in the open-label, single-arm, phase 2 KEYNOTE-158 study. Lancet Respir Med 2021; 9:613–621. 33836153 10.1016/S2213-2600(20)30515-4

[52] Chien KS Kim K Nogueras-Gonzalez GM. Phase II study of azacitidine with pembrolizumab in patients with intermediate-1 or higher-risk myelodysplastic syndrome. Br J Haematol 2021; 195:378–387. 34340254 10.1111/bjh.17689

[53] McDermott DF Lee J-L Ziobro M. Open-label, single-arm, phase II study of pembrolizumab monotherapy as first-line therapy in patients with advanced non-clear cell renal cell carcinoma. J Clin Oncol 2021; 39:1029–1039. 33529058 10.1200/JCO.20.02365 PMC8078262

[54] Puig N Corchete-Sánchez LA Pérez-Morán JJ. Pembrolizumab as consolidation strategy in patients with multiple myeloma: results of the GEM-pembresid clinical trial. Cancers (Basel) 2020; 12:3615. 33287189 10.3390/cancers12123615 PMC7761692

[55] Matsuoka H Hayashi T Takigami K. Correlation between immune-related adverse events and prognosis in patients with various cancers treated with anti PD-1 antibody. BMC Cancer 2020; 20:656. 32664888 10.1186/s12885-020-07142-3 PMC7362440

[56] Tasaka Y Honda T Nishiyama N. Noninferior clinical outcomes of immune checkpoint inhibitors in nonsmall cell lung cancer patients with interstitial lung disease. Lung Cancer 2021; 155:120–126. 33798901 10.1016/j.lungcan.2021.03.014

[57] Dobre IA Frank AJ D'Silva KM. Outcomes of patients with interstitial lung disease receiving programmed cell death 1 inhibitors: a retrospective case series. Clinical Lung Cancer 2021; 22:e738–e744. 33663958 10.1016/j.cllc.2021.01.014 PMC8829839

[58] Rogiers A Pires da Silva I Tentori C. Clinical impact of COVID-19 on patients with cancer treated with immune checkpoint inhibition. J Immunother Cancer 2021; 9:e001931. 33468556 10.1136/jitc-2020-001931 PMC7817383

[59] Hijmering-Kappelle LBM Hiltermann TJN Bensch F. Safety and efficacy of extended interval dosing for immune checkpoint inhibitors in non-small cell lung cancer during the COVID-19 pandemic. Clin Lung Cancer 2022; 23:143–150. 35034861 10.1016/j.cllc.2021.12.005 PMC8704727

[60] Chuzi S Tavora F Cruz M. Clinical features, diagnostic challenges, and management strategies in checkpoint inhibitor-related pneumonitis. Cancer Manag Res 2017; 9:207–213. 28652812 10.2147/CMAR.S136818 PMC5476791

[61▪] Kowalski B Valaperti A Bezel P. Analysis of cytokines in serum and bronchoalveolar lavage fluid in patients with immune-checkpoint inhibitor-associated pneumonitis: a cross-sectional case-control study. J Cancer Res Clin Oncol 2021; 148:1711–1720. 34347128 10.1007/s00432-021-03750-z PMC9189083

[62] Tabchi S Messier C Blais N. Immune-mediated respiratory adverse events of checkpoint inhibitors. Curr Opin Oncol 2016; 28:269–277. 27138570 10.1097/CCO.0000000000000291

[63] Peters S Gettinger S Johnson ML. Phase II trial of atezolizumab as first-line or subsequent therapy for patients with programmed death-ligand 1-selected advanced nonsmall-cell lung cancer (BIRCH). J Clin Oncol 2017; 35:2781–2789. 28609226 10.1200/JCO.2016.71.9476 PMC5562171

[64] Fehrenbacher L Spira A Ballinger M. Atezolizumab versus docetaxel for patients with previously treated nonsmall-cell lung cancer (POPLAR): a multicentre, open-label, phase 2 randomised controlled trial. Lancet 2016; 387:1837–1846. 26970723 10.1016/S0140-6736(16)00587-0

[65] Rittmeyer A Barlesi F Waterkamp D. Atezolizumab versus docetaxel in patients with previously treated nonsmall-cell lung cancer (OAK): a phase 3, open-label, multicentre randomised controlled trial. Lancet 2017; 389:255–265. 27979383 10.1016/S0140-6736(16)32517-X PMC6886121

[66] Kaufman HL Russell J Hamid O. Avelumab in patients with chemotherapy-refractory metastatic Merkel cell carcinoma: a multicentre, single-group, open-label, phase 2 trial. Lancet Oncol 2016; 17:1374–1385. 27592805 10.1016/S1470-2045(16)30364-3 PMC5587154

[67] Balestra R Benzaquen S Wang J. Sarcoidosis-like granulomatous lung reaction associated with antiprogrammed death receptor-1 ligand therapy. Ann Am Thorac Soc 2017; 14:296–299. 10.1513/AnnalsATS.201611-863LE 28146383

[68] Antonia SJ Villegas A Daniel D. Durvalumab after chemoradiotherapy in stage III non–small-cell lung cancer. N Engl J Med 2017; 377:1919–1929. 28885881 10.1056/NEJMoa1709937

[69] Garassino MC Cho B-C Kim J-H. Durvalumab as third-line or later treatment for advanced nonsmall-cell lung cancer (ATLANTIC): an open-label, single-arm, phase 2 study. Lancet Oncol 2018; 19:521–536. 29545095 10.1016/S1470-2045(18)30144-X PMC7771363

[70] Wolchok JD Kluger H Callahan MK. Nivolumab plus ipilimumab in advanced melanoma. N Engl J Med 2013; 369:122–133. 23724867 10.1056/NEJMoa1302369 PMC5698004

[71] Larkin J Chiarion-Sileni V Gonzalez R. Combined nivolumab and ipilimumab or monotherapy in untreated melanoma. N Engl J Med 2015; 373:23–34. 26027431 10.1056/NEJMoa1504030 PMC5698905

[72] Postow MA Chesney J Pavlick AC. Nivolumab and ipilimumab versus ipilimumab in untreated melanoma. N Engl J Med 2015; 372:2006–2017. 25891304 10.1056/NEJMoa1414428 PMC5744258

[73] Antonia SJ López-Martin JA Bendell J. Nivolumab alone and nivolumab plus ipilimumab in recurrent small-cell lung cancer (CheckMate 032): a multicentre, open-label, phase 1/2 trial. Lancet Oncol 2016; 17:883–895. 27269741 10.1016/S1470-2045(16)30098-5

[74] Cui PF Ma JX Wang FX. Pneumonitis and pneumonitis-related death in cancer patients treated with programmed cell death-1 inhibitors: a systematic review and meta-analysis. Ther Clin Risk Manag 2017; 13:1259- L 1271. 29026313 10.2147/TCRM.S143939 PMC5626381

[75] Reuss JE Kunk PR Stowman AM. Sarcoidosis in the setting of combination ipilimumab and nivolumab immunotherapy: a case report & review of the literature. J Immunother Cancer 2016; 4:94. 28031822 10.1186/s40425-016-0199-9 PMC5168862

